# Effect of long work hours and shift work on high-sensitivity C-reactive protein levels among Korean workers

**DOI:** 10.5271/sjweh.3933

**Published:** 2021-03-31

**Authors:** Wanhyung Lee, Seong-Kyu Kang, Won-Jun Choi

**Affiliations:** Department of Occupational and Environmental Medicine, Gil Medical Center, Gachon University College of Medicine, Incheon, Republic of Korea

**Keywords:** cardiovascular disease, hsCRP, inflammation, KNHANES, Korea, shift worker, working hour

## Abstract

**Objective:**

We aimed to investigate the association between low-grade inflammation as indicated by high-­sensitivity C-reactive protein (hsCRP) level and organizational factors, such as work hours and shift work.

**Methods:**

We evaluated 7470 young and middle-aged workers who participated in the Korea National Health and Nutrition Examination Surveys from 2015–2018. Work hours were determined from self-reported questionnaires. Shift work was defined as a non-daytime fixed work schedule. An interaction effect between shift work and long work hours on the hsCRP level was estimated using relative excess risk due to interaction (RERI) and attributable proportion (AP) with 95% confidence intervals (CI).

**Results:**

Increased hsCRP levels were prevalent in 25.2% of the study population. There was a significant association between long work hours and increased hsCRP, especially among middle-aged men [odds ratio (OR) 1.50 (95% CI 1.20–1.87) for moderately increased hsCRP and OR 1.62 (95% CI 1.14–2.30) for highly increased hsCRP]. There was a significant interaction effect between long work hours and shift work on increased hsCRP among middle-aged workers. The RERI were 0.03 (95% CI 0.02–0.04) and 0.56 (95% CI 0.45–0.68) among middle-aged men and women, respectively. The AP were 0.02 (95% CI 0.01–0.03) and 0.36 (95% CI 0.31–0.40) among middle-aged men and women, respectively.

**Conclusions:**

There was no significant association between shift work and the level of hsCRP. Long work hours were related to low-grade inflammatory processes, but only in middle-aged workers. There was an interaction effect between long work hours and shift work for increased hsCRP, especially in middle-aged women.

Although an adequate systemic inflammatory response is essential for successful recovery from injuries and infections, chronic low-grade or subclinical inflammation may lead to health problems (1). Several reports have described the relationship between inflammation and cardiovascular diseases (CVD) (2), arrhythmias (3), stroke (4) and neurological disorders (5). A complex inflammatory response mediates the pathogenesis of atherosclerosis, which is the underlying pathology of CVD (6). Among numerous biomarkers related to systemic inflammation, the high-sensitivity C-reactive protein (hsCRP) level, which is easily accessible in the clinical setting (7), is associated with the risk of CVD and other vascular pathologic changes (8, 9).

Occupational factors, such as working hours and shift work, may be associated with various health problems (10, 11). The disruption of the circadian rhythm, exposure to artificial light, psychological stress, and other social and lifestyle factors associated with work patterns can promote chronic systematic inflammation (12–14). Factors associated with organizational work may also influence systemic inflammation. In a recent review, Virtanen & Kivimäki (15) concluded that long working hours were a risk factor for CVD. The plausible pathways leading from shift work to CVD are related to psychosocial, behavioral, and physiological mechanisms (16). Shift work may cause physiological stresses, such as inflammation, blood coagulation, and altered cardiac autonomic functions, which are related to atherosclerotic changes. Puttonen et al (17) reported that irregular working hours, such as 2- and 3-shift work schedules, were associated with an increased risk of inflammation among airline employees. Results of other studies indicate that shift work is associated with elevated hsCRP levels among female workers (18) and a higher prevalence of hypertension among male workers with elevated serum ferritin levels (19). However, information on the interactive effects of work-related factors, such as long working hours and shift work, on systemic inflammation in the working population is still scarce.

Thus, this study aimed to investigate the association between hsCRP and organizational factors such as working hours and shift work in a representative sample of Korean workers.

## Methods

### Study design and data collection

We used data from the Korea National Health and Nutrition Examination Surveys (KNHANES), 2015–2018, that comprise a series of nationally representative population-based surveys on the health and nutritional status of Korean citizens conducted by the Korea Centers for Disease Control and Prevention. The KNHANES were based on self-questionnaires that gather information on different aspects such as demographics, socioeconomic status, dietary habits, and medical history. The surveys were conducted by a trained interviewers’ assistant. Blood and urine sampling and anthropometric examination are also performed by trained professionals either at the home of respondents or in mobile examination centers (20). All KNHANES data are publicly available at the KNHANES website (http://knhanes.cdc.go.kr).

The participants of the KNHANES are identified annually through systematic sampling of Korean citizens using multistage clusters based on age, sex, and household registries. A total of 31 649 participants were included in the 2015–2018 KNHANES (7380 in 2015; 8150 in 2016; 8127 in 2017; and 7992 in 2018).

**Figure 1:**
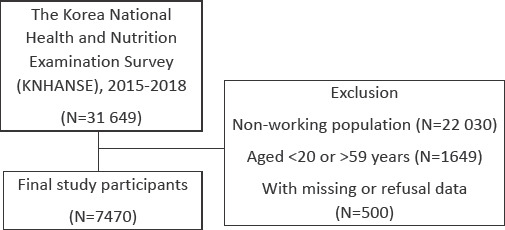
Participant inclusion flowchart.

The present study evaluated young and middle-aged workers. We excluded 22 030 individuals of the non-working population, 1649 individuals aged <20 or >59 years, and 500 individuals with missing data or who refused to share data. In total, 7470 participants were included in the analysis. The participant inclusion flowchart is shown in figure 1.

### Shift work and long work hours

Shift work was defined as a non-daytime fixed work schedule identified with the question “Do you mainly work weekly (06:00–18:00 hours)? Or do you work during other time slots?” An answer other than “daytime fixed working schedule (06:00–18:00 hours)” indicated a shift-work schedule. The shift-work schedule included evening (14:00–24:00 hours) work, night-time (21:00–08:00 hours) work, 24-hour shift, and split or irregular shifts.

Working hours were based on the total number of hours worked per week ascertained from the self-reported questionnaire that included the following question: “on average, excluding meal times, how many hours do you work at your job per week including overtime?”

The definitions of working schedules and long working hours reflect social, cultural, or economic circumstances and are changeable. We defined long working hours on the basis of the Korean labor standards and descriptions in previous studies. The Labor Standard Act in Korea allows weekly working hours of up to 52 hours, although the weekly working hours can be extended with the agreement of all parties (21). Previous studies from Korea indicated that if the work hours per week exceed 52 hours, the risk of illness because of long working hours increased (22, 23). Thus, we dichotomously categorized work hours into two groups on the basis of the responses “No” and “Yes” to long working hours (weekly work hours ≤52 and >52, respectively).

### High-sensitivity C-reactive protein

The hsCRP level was determined from blood samples collected in 3-mL ethylenediaminetetraacetic acid-coated tubes (BD Vacutainer, Franklin Lakes, NJ, USA), stored at 2–8°C in refrigerated containers, and analyzed within 24 hours of sample collection. The hsCRP level was measured using an immunoturbidimetric method (Cobas, Roche, Germany). Human CRP agglutinates with latex particles coated with monoclonal anti-CRP antibodies, and the aggregates obtained were quantified turbidimetrically. The Food and Drug Administration has approved this method for clinical use in Korea and other developed countries (24, 25). In this study, the participants were categorized into three groups based on the hsCRP level in accordance with the criteria of the American Heart Association and Centers for Disease Control and Prevention: normal <1.0; moderately increased 1.0–3.0; and highly increased >3.0 mg/L (26).

### Other covariates

We used age, sex, educational status. and household income level as socioeconomic variables. The educational level was stratified into three levels: graduate to middle school, high school, and college or higher. The household income level was categorized by quartiles [lowest (quartile 1) to highest (quartile 4)] based on the yearly household income level. Past smoking indicated smokers who had stopped tobacco smoking for ≥1 month. Individuals who had smoked <100 cigarettes in their lifetime were placed in the “none” category. Moderate alcohol drinking for women and men was defined as the consumption of <5 and <7 glasses of alcohol ≤2 times per week, respectively. Severe alcohol drinking was classified as the consumption of more than the moderate alcohol intake level. Metabolic syndrome was diagnosed according to the recommendations of the International Diabetes Federation (27) and was defined as the presence of ≥3 of the following 5 abnormalities: (i) central obesity (waist circumference >90 cm among men or >80 cm among women); (ii) hypertension (blood pressure ≥130/85 mmHg or antihypertensive drug treatment); (iii) hyperglycemia (fasting glucose level of serum ≥100 mg/dL or use of antidiabetic medication); (iv) high triglyceride (TG) levels (TG ≥150 mg/dL or drug treatment for dyslipidemia); or (v) low high-density lipoprotein cholesterol (HDL-C) levels (<40 mg/dL among men and <50 mg/dL among women).

### Statistical analysis

The differences in general characteristics according to shift work or long work hours were calculated for each variable using the chi-square test. Odds ratios (OR) and 95% confidence intervals (CI) were calculated using logistic regression models to evaluate the association between the hsCRP level and shift work or long work hours with sex and age group stratification. We calculated the age-standardized prevalence ratio of abnormal hsCRP (>1.0 mg/L) according to work conditions with reference to the same-aged general population in the KNHANES. The interaction effect between shift work and long work hours on hsCRP level was estimated with the P-value. In subgroup analysis with sex and age stratification, an interaction effect was demonstrated based on the P-value, and a relative excess risk due to interaction (RERI) and attributable proportion (AP) were represented by the 95% CI. All of the interaction effects were estimated with “no long work hours” and “no shift work” group as a reference. All statistical analyses were performed using SAS (version 9.4; SAS Institute, Cary, NC, USA). Two-tailed P-values <0.05 were considered statistically significant.

## Results

The baseline characteristics according to hsCRP levels are presented in table 1. The prevalence of extremely high and moderately high hsCRP levels was higher among men than women. A lower household income level was significantly associated with a higher prevalence of increased hsCRP levels. Current smokers and participants with metabolic syndrome showed significantly higher prevalence of increased hsCRP levels. The long work hours group also had a higher prevalence of increased hsCRP levels.

**Table 1:** Participant characteristics according to high-sensitivity C-reactive protein (hsCRP) levels. [MetS=metabolic syndrome.]

Characteristics	Total participants	hsCRP (mg/L)			P-value

		Normal (<1.0)	Moderately increased (1.0–3.0)	Highly increased (>3.0)	
			
	N (%)	N (%)	N (%)	N (%)	
Overall	7470	5581	1397	492	
Sex					<0.0001
Men	3774 (50.5)	2687 (71.1)	806 (21.4)	281 (7.5)	
Women	3696 (49.5)	2894 (78.3)	591 (16.0)	211 (5.7)	
Age (years)					0.7118
20–39	3176 (42.5)	2389 (75.2)	571 (18.0)	216 (6.8)	
40–59	4294 (57.5)	3192 (74.3)	826 (19.2)	276 (6.5)	
Education level					0.3780
Middle school	595 (8.0)	430 (72.3)	132 (22.2)	33 (5.5)	
High school	2858 (38.3)	2119 (74.1)	555 (19.5)	184 (6.4)	
≥College	4017 (53.7)	3032 (75.5)	710 (17.7)	275 (6.8)	
Household income (quartile)					<0.0001
1^st^	432 (5.7)	314 (72.7)	85 (19.7)	33 (7.6)	
2^nd^	1538 (20.6)	1097 (71.3)	318 (20.7)	123 (8.0)	
3^rd^	2530 (33.9)	1889 (74.7)	480 (19.0)	161 (6.3)	
4^th^	2970 (39.8)	2281 (76.8)	514 (17.3)	175 (5.9)	
Smoking					<0.0001
None	4270 (57.2)	3307 (77.5)	709 (16.6)	254 (5.9)	
Past	1461 (19.6)	1061 (72.6)	300 (20.5)	100 (6.9)	
Current	1739 (23.2)	1213 (69.8)	388 (22.3)	138 (7.9)	
Drinking					0.3420
None	1090 (14.6)	802 (73.6)	210 (19.2)	78 (7.2)	
Moderate	5266 (70.5)	3982 (75.6)	395 (18.1)	331 (6.3)	
Severe	1114 (14.9)	797 (71.5)	234 (21.0)	83 (7.5)	
MetS					<0.0001
No	6020 (80.6)	4766 (79.2)	925 (15.4)	329 (5.4)	
Yes	1450 (19.4)	815 (56.2)	472 (32.6)	163 (11.2)	
Shift work					0.3973
No	6205 (83.1)	4627 (72.6)	1162 (18.7)	416 (6.7)	
Yes	1265 (16.9)	954 (75.4)	235 (18.6)	76 (6.0)	
Long work hours					0.0004
No	6314 (84.5)	4770 (75.5)	1141 (18.1)	403 (6.4)	
Yes	1156 (15.5)	811 (70.2)	256 (22.1)	89 (7.7)	

Table 2 presents the results of the logistic regression analysis for the risk of increased hsCRP levels with respect to shift work or long work hours based on sex and age group stratification. Significantly elevated risks of moderately and highly increased hsCRP levels were identified among men in the long work hours group after adjustments for age, educational level, income level, smoking, alcohol consumption, and metabolic syndrome [OR 1.21 (95% CI 1.02–1.44) and OR 1.33 (95% CI 1.01–1.76), respectively]. After sex and age group stratification, the association between long work hours and increased hsCRP levels was enhanced among middle-aged men [40–59 years: OR 1.50 (95% CI 1.20–1.87) and OR 1.62 (95% CI 1.14–2.30), respectively]. However, there was no significant association between shift work and increased hsCRP levels.

**Table 2:** Association between high-sensitivity C-reactive protein (hsCRP) level and working condition on the logistic regression analyses. All models are adjusted for age, educational level, income level, smoking, alcohol consumption, and metabolic syndrome. **Numbers in bold** indicate statistical significance. [OR=odds ratio; 95% CI=95% confidence interval]

	N	hsCRP ≥1.0 mg/L	hsCRP >3.0 mg/L
	
		OR (95% CI)	OR (95% CI)
Total participants	7470		
Shift work			
No	6205	1.00 (Reference)	1.00 (Reference)
Yes	1265	0.99 (0.86–1.15)	0.91 (0.70–1.17)
Long work hours			
No	6314	1.00 (Reference)	1.00 (Reference)
Yes	1156	**1.18 (1.03–1.37**)	1.15 (0.90–1.47)
Men	3774		
Shift work			
No	3145	1.00 (Reference)	1.00 (Reference)
Yes	629	0.91 (0.74–1.11)	0.92 (0.65–1.30)
Long work hours			
No	2944	1.00 (Reference)	1.00 (Reference)
Yes	830	**1.21 (1.02–1.44**)	**1.33 (1.01–1.76**)
Women	3696		
Shift work			
No	3060	1.00 (Reference)	1.00 (Reference)
Yes	636	1.10 (0.70–1.36)	0.88 (0.59–1.29)
Long work hours			
No	3370	1.00 (Reference)	1.00 (Reference)
Yes	326	0.99 (0.74–1.32)	0.63 (0.35–1.12)
Men (20–39 years)	1631		
Shift work			
No	1341	1.00 (Reference)	1.00 (Reference)
Yes	290	0.92 (0.67–1.25)	1.08 (0.65-1.81)
Long work hours			
No	1275	1.00 (Reference)	1.00 (Reference)
Yes	356	0.84 (0.63–1.11)	0.94 (0.59–1.52)
Women (20–39 years)	1545		
Shift work			
No	1297	1.00 (Reference)	1.00 (Reference)
Yes	248	1.10 (0.76–1.59)	0.64 (0.33–1.23)
Long work hours			
No	1432	1.00 (Reference)	1.00 (Reference)
Yes	113	1.28 (0.78–2.09)	0.44 (0.15–1.28)
Men (40–59 years)	2143		
Shift work			
No	1804	1.00 (Reference)	1.00 (Reference)
Yes	339	0.89 (0.68–1.17)	0.78 (0.49–1.26)
Long work hours			
No	1669	1.00 (Reference)	1.00 (Reference)
Yes	**474**	**1.50 (1.20–1.87)**	**1.62 (1.14–2.30)**
Women (40–59 years)	2151		
Shift work			
No	1763	1.00 (Reference)	1.00 (Reference)
Yes	388	1.14 (0.87–1.50)	1.11 (0.68–1.83)
Long work hours			
No	1938	1.00 (Reference)	1.00 (Reference)
Yes	213	0.90 (0.63–1.29)	0.78 (0.38–1.59)

Table 3 presents the results of the interaction effect of long working hours and shift work on the risk of increased hsCRP levels (≥1.0 mg/L). Overall, there was no interaction between long working hours and shift work with the hsCRP level (P-value for interaction = 0.1648). After sex-specific stratification, the strata with only women showed a statistically significant interaction effect on increased hsCRP levels (P-value for interaction = 0.0001).

**Table 3:** Interaction effect of long work hours and shift work on elevated high-sensitivity C-reactive protein level (hsCRP ≥1.0 mg/L) [SPR=age-standardized prevalence ratio; CI=confidence interval]

	Long work hours		P-value for interaction ^a^

	No, SPR (95% CI)	Yes, SPR (95% CI)	
Total participants			0.1648
Shift work			
No	1.00 (0.95–1.06)	1.24 (1.10–1.38)	
Yes	0.98 (0.85–1.11)	1.21 (0.91–1.51)	
Men			0.9584
Shift work			
No	1.03 (0.95–1.10)	1.21 (1.05–1.37)	
Yes	0.95 (0.77–1.13)	1.05 (0.75–1.35)	
Women			0.0001
Shift work			
No	0.96 (0.88–1.04)	1.01 (0.75–1.26)	
Yes	1.02 (0.83–1.20)	1.38 (0.70–2.05)	

a Interaction effects were estimated with respect to no long work hours and no shift work group as a reference.

**Figure 2:**
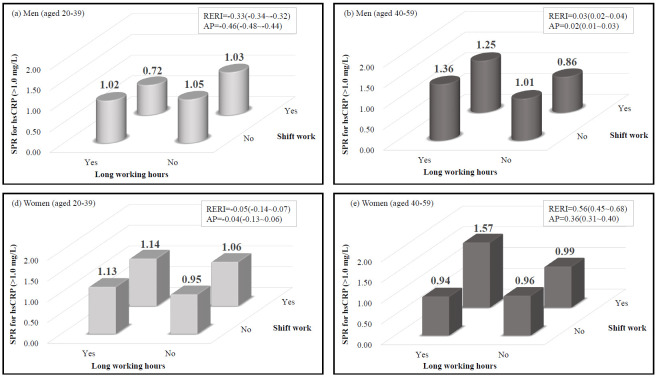
Results of interaction analysis of long work hours and shift work on increased high-sensitivity C-reactive protein (hsCRP) level with sex and age group stratification. Interaction effects were estimated with respect to no long work hours and no shift work group as a reference. [SPR=age-standardized prevalence ratio; RERI=relative excess risk due to interaction with 95% confidence interval; AP= attributable proportion with 95% confidence interval].

Figure 2 shows the results of the interaction analysis of long work hours with shift work and the influence on higher hsCRP level with regard to sex and age group stratification. There was an interaction effect between long work hours and shift work on increased hsCRP levels among middle-aged men and women. The RERI were 0.03 (95% CI 0.02–0.04) and 0.56 (95% CI 0.45–0.68) among middle-aged men and women, respectively. The AP were 0.02 (95% CI 0.01–0.03) and 0.36 (95% CI 0.31–0.40) among middle-aged men and women, respectively. However, only middle-aged women showed a statistically significant result (P-value for interaction = 0.0365)

## Discussion

This study investigated the association of work conditions, such as long work hours and shift work, with the hsCRP level, which was considered a systematic proinflammatory factor. Male workers with long work hours had a higher likelihood of increased hsCRP levels. Despite the attenuation of the relationship between the risk of increased hsCRP levels and shift-work schedule after controlling for confounders, we found a significant interaction for an increased prevalence of elevated hsCRP levels between long work hours and the shift work schedule, especially among the middle-aged working population.

We found that long work hours were associated with elevated hsCRP levels, consistent with the findings of previous studies that showed a close association between long work hours and elevated hsCRP levels, especially among older workers (28). Long work hours could lead to sleep disturbance that is, in turn, directly associated to a lack of time for physical and neuropsychiatric recovery (21, 29). Previous studies have indicated that sleep disturbance is associated with increased levels of markers of systemic inflammation, including hsCRP (30). Extended work hours could activate the stress-response system that would influence the hypothalamic–pituitary–adrenal axis to increase the levels of glucocorticoids, which is considered a response to increased systematic inflammation (31). Moreover, a higher prevalence of smoking, obesity, and other adverse behaviors has been reported in those who work long hours (32).

A recent study showed that shift work influenced some immunological biomarkers (33). Thus, we hypothesized that hsCRP is also associated with shift work; however, we found no significant association between shift work and elevated hsCRP levels. This finding is inconsistent with that of an earlier report (34). However, the interaction between long work hours and shift work and elevated hsCRP levels was statistically significant. This result is consistent with previous studies that reported that the association between shift work and elevated inflammatory indices is markedly evident among those with long work hours or those working in prolonged shifts (35, 36). Zeler et al (35) investigated the oxidative and inflammatory status according to physical workloads of employees. They found that the levels of various inflammatory markers, including total peroxides, total antioxidant capacity, adrenocorticotropic hormone, oxidative stress index, and hsCRP, were significantly higher among heavy workers (slaughterhouse employees) than among office workers, especially those who worked longer hours (ie, 12- versus 8-hour shifts).

Buyukhatipoglu et al (36) also reported increased oxidative stress indices after prolonged work hours among healthcare workers who were on 24-hour on-call shifts compared with non-healthcare staff who worked 8-hour shifts. Thus, the hypothesis that both long work hours and shift work are more likely to induce systemic inflammation with an interaction effect, as shown in this study, seems feasible. A plausible explanation for this is the alterations in circadian rhythms that exacerbate chronic systemic inflammation by triggering proinflammatory activation of macrophages and inflammatory signaling pathways (37).

It remains unclear why the interaction effect was only significant among women, although sex differences in the circadian system may be a possible explanation. Duffy et al (38) reported that the intrinsic circadian period was shorter among women than men, and a greater proportion of women had intrinsic circadian periods <24 hours (38). This shorter intrinsic circadian period leads to circadian misalignment associated with alteration in physiologic processes, including elevated levels of markers of systemic inflammation. Wright et al (39) observed significantly increased CRP levels in the circadian misalignment group and decreased CRP levels in the synchronized group after weeks of circadian entrainment.

There may be another possible explanation considering the difference in total working hours between men and women. The total working hours of women may be longer than those of men because women do more domestic labor. The Statistics Korea 2019 nationwide Time Use Survey showed that men (husbands) of dual-income households spent 8 hours and 46 minutes for inevitable daily activities, including 5 hours and 50 minutes for work and 54 minutes for domestic labor, whereas women (wives) of dual-income households spent 9 hours and 24 minutes for inevitable daily activities, including 4 hours and 37 minutes for work and 3 hours and 7 minutes for domestic labor (40). Men and women in single-income households spent very similar time for inevitable daily activities compared with those in dual-income households. Although only average statistics are available and such statistics cannot be directly linked to this study data, women apparently do more domestic labor than men, even when they have a job. Women who work long hours with shift work may have less time for rest than men with the same work characteristics because women do more housework. This may be one of the possible explanations why the interaction effect between long work hours and shift work on the increase of hsCRP levels was found only among women.

Given the nature of chronic diseases, one of the best approaches to prevent CVD is primary prevention. Emphasis should be placed on controlling risk factors and reducing unsafe behaviors and conditions to prevent CVD. From this viewpoint, reducing work hours or maintaining an appropriate level of work hours for young and middle-aged workers may be helpful to reduce the risk of diseases that occur due to chronic inflammation. Special attention should be focused on shift workers to emphasize the need to control other risk factors for the prevention of diseases when shift work is inevitable.

The present work contributes to the existing knowledge base of the association between work conditions and inflammation by providing an interaction analysis with sex- and age group-stratified analysis that was adjusted for confounders. In support of the evidence from previous studies that were conducted with relatively small sample sizes, we report a significant association between long work hours and shift work and increased hsCRP levels in a large representative sample of Korean workers.

This study had some limitations, the major being its cross-sectional design. This made it difficult to determine a causality association between work conditions and hsCRP. Further longitudinal studies are required to ascertain the causality in this regard. Moreover, we could not describe other work condition-related factors of inflammation besides the work hours and working schedule due to a lack of information about workplace hazardous factors in the KNHANES. Systematic inflammation could have been increased because of exposure to various hazardous factors such as organic chemicals, dust, or stress at the workplace. The findings of this present work cannot be extrapolated to all work conditions in the general population. We could not describe the duration of career or the duration of exposure to shift work schedule or long work hours associated to the level of hsCRP because of the lack of information on these aspects in the KNHANES. Further studies are needed to identify the dose–response relationship between shift work or long work hours and the risk of elevated hsCRP levels.

In conclusion, long work hours were significantly associated with increased hsCRP levels among middle-aged men. Shift work itself was not a risk factor for low-grade inflammation. However, there was a significant interaction effect between long work hours and shift work on low-grade inflammation, especially among middle-aged women. Overall, working hours were related to low-grade inflammatory processes, but only among middle-aged workers.
